# Identification and validation of a siglec-based and aging-related 9-gene signature for predicting prognosis in acute myeloid leukemia patients

**DOI:** 10.1186/s12859-022-04841-5

**Published:** 2022-07-19

**Authors:** Huiping Shi, Liang Gao, Weili Zhang, Min Jiang

**Affiliations:** 1 Soochow University Medical College, Suzhou, Jiangsu People’s Republic of China; 2 Institutes of Biology and Medical Sciences, Soochow University, Suzhou, Jiangsu People’s Republic of China; 3 Department of Gastroenterology, Xiangcheng People’s Hospital, Suzhou, 215131 People’s Republic of China; 4 Department of Oncology, The First Affiliated Hospital of Soochow University, Suzhou, 215006 People’s Republic of China

**Keywords:** Siglec, Acute myeloid leukemia, Aging, The cancer genome atlas, Drug resistance, Prognostic model

## Abstract

**Background:**

Acute myeloid leukemia (AML) is a group of highly heterogenous and aggressive blood cancer. Despite recent progress in its diagnosis and treatment, patient outcome is variable and drug resistance results in increased mortality. The siglec family plays an important role in tumorigenesis and aging. Increasing age is a risk factor for AML and cellular aging contributes to leukemogenesis via various pathways.

**Methods:**

The differential expression of the siglec family was compared between 151 AML patients and 70 healthy controls, with their information downloaded from TCGA and GTEx databases, respectively. How siglec expression correlated to AML patient clinical features, immune cell infiltration, drug resistance and survival outcome was analyzed. Differentially expressed genes in AML patients with low- and high-expressed siglec9 and siglec14 were analyzed and functionally enriched. The aging-related gene set was merged with the differentially expressed genes in AML patients with low and high expression of siglec9, and merged genes were subjected to lasso regression analysis to construct a novel siglec-based and aging-related prognostic model. The prediction model was validated using a validation cohort from GEO database (GSE106291).

**Results:**

The expression levels of all siglec members were significantly altered in AML. The expression of siglecs was significantly correlated with AML patient clinical features, immune cell infiltration, drug resistance, and survival outcome. Based on the differentially expressed genes and aging-related gene set, we developed a 9-gene prognostic model and decision curve analysis revealed the net benefit generated by our prediction model. The siglec-based and aging-related 9-gene prognostic model was tested using a validation data set, in which AML patients with higher risk scores had significantly reduced survival probability. Time-dependent receiver operating characteristic curve and nomogram were plotted and showed the diagnostic accuracy and predictive value of our 9-gene prognostic model, respectively.

**Conclusions:**

Overall, our study indicates the important role of siglec family in AML and the good performance of our novel siglec-based and aging-related 9-gene signature in predicting AML patient outcome.

**Supplementary Information:**

The online version contains supplementary material available at 10.1186/s12859-022-04841-5.

## Background

Acute myeloid leukemia (AML) is a group of malignant hematological cancer that originates from the myeloid lineage cells. The rapid progression, great heterogenicity and frequent mutation of AML result in its high mortality. Current molecular diagnosis has greatly increased our understanding of AML and laid a foundation for individualized and targeted therapy. Meanwhile, great progress has been made in AML treatment, including the use of novel kinase inhibitors, monoclonal antibodies, and chimeric antigen receptor (CAR)-T cell therapy [1]. However, some key issues are still limiting AML treatment efficacy, such as relapse, drug resistance and side effects. To overcome these bottlenecks, specific molecular targets are needed to improve AML risk-stratification, combat drug resistance and reduce off-target effects.

Siglecs are a family of sialic acid binding IgG-like lectins. Currently, 14 members of the siglec family have been discovered in human, with each of them displaying varying expression patterns, molecular structures and sialoside preferences [2]. Siglecs regulate immune function and participate in various human diseases including AML. Novel strategies are being designed to harness siglecs for improving AML therapy. CD33 (i.e. siglec3) is a well-established target in AML and forms the basis of multiple AML therapies, including the immunoconjugate gemtuzumab ozogamicin [3], CAR-T immunotherapy [4], and bispecific antibodies [5]. Additionally, CAR-T cells targeting siglec6 was developed to eliminate leukemia cells with limited off-target effects due to the deficiency of siglec6 on normal hematopoietic stem and progenitor cells [6]. Recent studies also indicate that siglec9 is an inhibitory immune checkpoint in tumor [7, 8]. Endogenous immune response and therapeutic efficacy of tumor-targeting antibodies were inhibited in a humanized mouse model for siglec7/9. Blocking antibodies to siglec7/9 could reduce tumor burden and enhance antitumor immunity in mice [7]. Siglec-7 has been shown to interact with the mucin-type glycoprotein CD43 on leukemia cells, which leads to immune inhibition. CD43 knockout or blockade in leukemia cells disrupted this interaction and reduced the siglec7-mediated immune inhibition [9]. These studies depict the great potential of using siglecs as novel molecular biomarkers and therapeutic targets in AML.

The risk of developing AML is increasing with aging, with about 75% of AML patients aged ≥ 60 years in the US [10]. Age is also correlated with AML prognosis as the incidence of multidrug resistance, unfavorable cytogenetics, and chromosome abnormalities increases in elderly AML patients [11]. Cellular aging is one of the major risk factors for leukemogenesis [12]. During hematopoietic cell aging, there are multiple cellular processes that contribute to AML pathogenesis, including somatic mutations, epigenetic reprogramming, mitochondria dysfunction, oxidative stress, and proteostasis disturbance [13, 14]. Siglec-E is the mouse orthologue of human siglec9. It is reported that mice lacking siglec-E had accelerated aging and reduced lifespan [15]. Siglec-E deficiency contributed to dysregulated ROS metabolism and subsequent damage to cellular DNA, proteins, and lipids, which were related to accelerated aging [15]. It remains unknown whether siglec9, the human orthologue of mouse siglec-E, is also involved in the human aging process and contributes to human aging-related disease. Due to these complex aging-related pathological changes in AML and the role of siglecs in aging, it’s of urgent necessity to establish a siglec- and aging-related risk stratification system for AML patients, which might reveal novel prognostic markers for improved AML patient outcome prediction.

In this study, we performed a comprehensive analysis to dissect the role of siglec family in AML clinical characteristics, immune cell infiltration, treatment resistance and patient outcome. In addition, the differentially expressed genes in AML patients with different levels of siglecs were merged with aging-related gene set to build a 9-gene prognostic model. The novel 9-gene model was tested in a validation data set and showed good performance in predicting AML patient outcome.

## Methods

### Data collection

The clinical and RNA-seq data from a total of 151 AML patients were downloaded from TCGA database (https://portal.gdc.cancer.gov/). This cohort consists of adult de novo AML patients and the sequencing was performed using whole blood samples [16]. For comparison, the RNA-seq data of 70 healthy controls were downloaded from GTEx (https://gtexportal.org/home/). RNA-seq data in the format of level 3 HTSeq-FPKM (fragments per kilobase per million) were converted to TPM (transcripts per million reads) format and log2 transformed.

The validation dataset (GSE106291) was downloaded from GEO database, which consists of 210 patients from the AMLCG-2008 study (NCT01382147) and 40 patients from the AMLG-1999 trial (NCT00266136) [17]. These cases were newly diagnosed AML patients, and samples for sequencing were bone marrow or peripheral blood mononuclear cells [18, 19]. The aging-related gene set was downloaded from the National Genomics Data Center [20] (Additional file 1: Table S1).

### Siglec expression comparison

The ggplot2 package (version 3.3.3) was used to analyze and visualize the differential expression of siglecs between normal controls and AML patients. The RNA-seq data in the format of TPM were download from UCSC XENA (https://xenabrowser.net/datapages/), which were originally from TCGA and GTEx databases and processed by Toil [21].

### Correlation between clinical features and siglec expression

The ggplot2 package (version 3.3.3) was used to analyze and visualize the differential expression of siglecs between AML subgroups, which were categorized by WBC count, PB blasts, BM blasts, NPM1 mutation, IDH1 R132 mutation and FLT3 mutation.

### Immune infiltration

The GSVA package (version 1.34.0) [22] was used to perform Spearman correlation analysis of siglec expression and immune cell infiltration (algorithm: ssGSEA). The analyzed immune cells included: activated DC (aDC); B cells; CD8 T cells; cytotoxic cells; DC; eosinophils; immature DC (iDC); macrophages; mast cells; neutrophils; NK CD56^bright^ cells; NK CD56^dim^ cells; NK cells; plasmacytoid DC (pDC); T cells; T helper cells; T central memory (Tcm); T effector memory (Tem); T follicular helper (Tfh); T gamma delta (Tgd); Th1 cells; Th17 cells; Th2 cells; Treg [23].

### Treatment resistance

The relationship between drug sensitivity and siglec expression was investigated using Gene Set Cancer Analysis (GSCA) [24]. The correlation analysis was performed based on data from the Genomics of Drug Sensitivity in Cancer (GDSC) database.

The ggplot2 package (version 3.3.3) was used to analyze and visualize the correlation between the expression levels of siglecs and PDCD-1 (PD-1) / CD274 (PD-L1) /CLTA4. Shapiro–Wilk test was used to check data normality. Pearson correlation analysis was used for analyzing parametric data and Spearman correlation analysis was used for analyzing nonparametric data.

### Receiver operating characteristic (ROC) curve analysis

The pROC package (version 1.17.0.1) was used for ROC analysis and the ggplot2 package (version 3.3.3) was used for visualization. The RNA-seq data in the format of TPM were download from UCSC XENA (https://xenabrowser.net/datapages/), which were originally from TCGA and GTEx databases and processed by Toil [21].

### Kaplan–Meier (KM) analysis

Survminer package (version 0.4.9) and survival package (version 3.2-10) were used for analysis of the overall survival data of AML patients with differential expression of siglecs [25]. AML patients were categorized into low and high expression groups according to the median expression level of the selected siglec.

### Differentially expressed gene (DEG) analysis

The DESeq2 package (version 1.26.0) [26] was used to identify the DEG in AML patients with low and high expression of selected siglec. RNA-seq data in the format of level 3 HTSeq-Counts were used. The cutoff for differential expression was |log2(FC)|> 1.5 and p.adj < 0.05. The ggplot2 package (version 3.3.3) was used to visualize the expression of most up-regulated and down-regulated genes in AML patients. Spearman correlation analysis was performed for the DEG and the selected siglec.

### Gene ontology (GO) and Kyoto encyclopedia of genes and genomes (KEGG) analysis

The clusterProfiler package (version 3.14.3) was used for enrichment analysis and the org.Hs.eg.db package (version 3.10.0) was used for ID conversion. The top 300 most differentially expressed genes in AML patients with low- and high-expression of siglec9 and siglec14 were used for GO/KEGG enrichment analysis [27]. The GO terms are classified as biological process (BP), cellular component (CC) and molecular function (MF).

### Gene set enrichment analysis (GSEA)

The clusterProfiler package (version 3.14.3) [27] was used for GSEA analysis, which analyzes the collective behavior of genes [28]. The reference gene set was c2.cp.v7.2.symbols.gmt (Curated). The information of 32,284 gene sets were derived from MSigDB Collections (https://www.gsea-msigdb.org/gsea/msigdb/collections.jsp#C2). Significant enrichment was defined as false discovery rate (FDR) < 0.25 and p.adj < 0.05.

### Construction of the siglec-based and aging-related prognostic model

The DEG identified between AML patients with low- and high-expressed siglec9 were merged with the aging-related gene set. The merged genes were subjected to least absolute shrinkage and selection operator (lasso) regression analysis using the glmnet package (version 4.1-2) and survival package (version 3.2-10). The lasso regression analysis was performed with tenfold cross validation. Lasso variable trace plot was used to visualize the lasso coefficient profile. The genes revealed by lasso regression analysis was used to calculate risk scores based on gene expression levels and corresponding lasso regression coefficients. The ggplot2 package (version 3.3.3) was used to visualize the risk score, time of survival and expression level of selected genes in AML patients.

### Validation of the prediction model

The survival probability of AML patients in low and high risk groups was analyzed using survminer package (version 0.4.9) and survival package (version 3.2-10). Time-dependent ROC curve analysis was performed using timeROC package (version 0.4) and ggplot2 package (version 3.3.3). Nomogram was performed using rms package (version 6.2-0) and survival package (version 3.2-10). Decision curve analysis (DCA) was performed using survival package (version 3.2-10) and stdca.R file.

### Statistics

Data analysis and visualization were performed using R (version 3.6.3). The R packages used in different analysis were specified in detail as shown above. The cutoff for differential expression was |log2(FC)|> 1.5 and p.adj < 0.05. The cutoff for GO/KEGG analyses was p.adj < 0.05 and qvalue < 0.2. For correlation analysis, Pearson correlation analysis was used for parametric data and Spearman correlation analysis was used for nonparametric data. *p* < 0.05 was considered statistically significant.

## Results

### Expression of the siglec family in AML patients and healthy controls

**Fig. 1:**
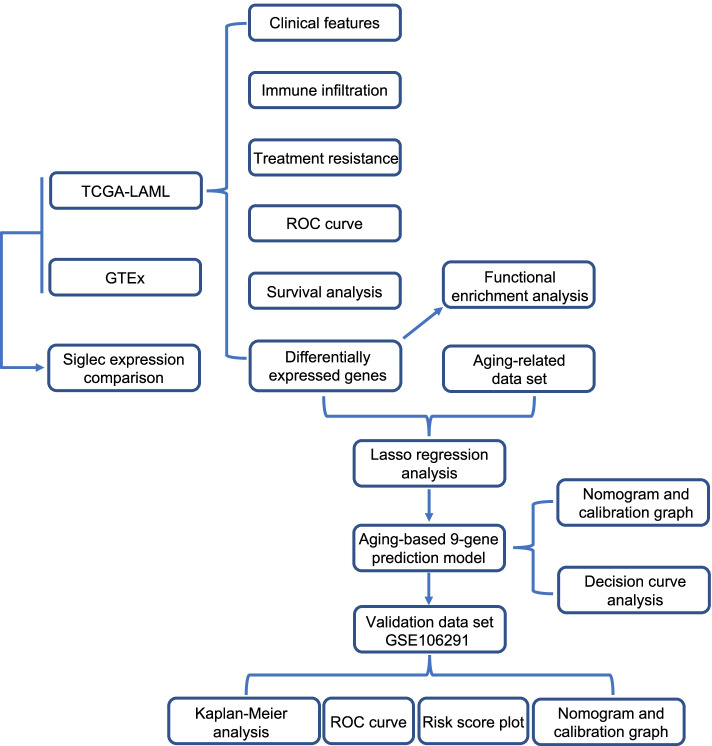
Flow chart showing the overall study design

**Fig. 2:**
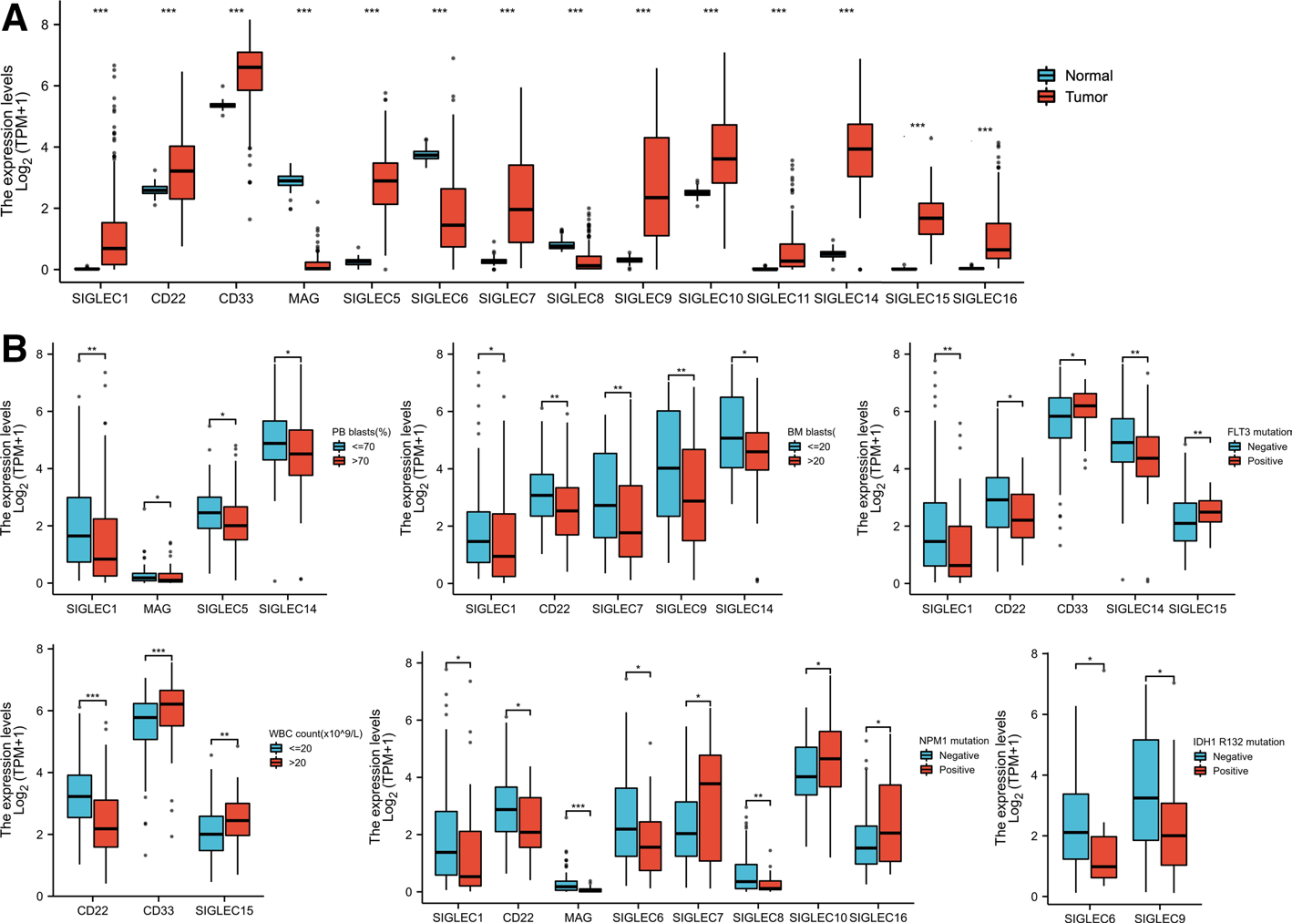
Expression the siglec family and its correlation with clinical features in AML. **A** The expression pattern of the siglec family in AML patients and healthy controls. **B** The differential expression of siglecs in AML sub-groups categorized by WBC count, PB blast, BM blast, FLT3 mutation, NPM1 mutation, IDH1 R132 mutation. *, *p* < 0.05; **, *p* < 0.01; ***, *p* < 0.001

The clinical features and RNA-seq data of 151 AML patients were downloaded from TCGA database, with a summary of patients’ general information shown in Additional file 2: Table S2. The healthy control data (n = 70) were obtained from GTEx database for comparison. The overall study design was shown in flow chart (Fig. 1). First, we compared the expression levels of 14 siglecs that are found in human between AML patients and healthy controls (Fig. 2A). Results showed that siglec1, CD22 (i.e. siglec2), CD33 (i.e. siglec3), siglec5, siglec7, siglec9, siglec10, siglec11, siglec14, siglec15 and siglec16 were significantly up-regulated in AML patients, whereas MAG (i.e. siglec4), siglec6 and siglec8 were significantly down-regulated in AML patients. Therefore, the expression levels of all siglec family members were significantly altered during AML pathogenesis, indicating their dysregulation and potential contribution to AML.

### Correlation between AML clinical characteristics and siglec expression

To gain insights into the relationship between siglec expression and AML patient features, we compared the siglec expression in AML subgroups categorized by WBC count, PB blasts, BM blasts, NPM1 mutation, IDH1 R132 mutation and FLT3 mutation (Fig. 2B). We found that expression levels of CD33 and siglec15 were significantly higher whereas CD22 expression was significantly lower in AML patients with WBC count > 20 × 10^9^/L. Siglec1, MAG, siglec5, and siglec14 expression levels were significantly reduced in AML patients with PB blast percentage > 70%. Expression levels of siglec1, CD22, siglec7, siglec9 and siglec14 were significantly higher in AML patients with BM blast percentage > 20%.

Mutations in NPM1, IDH1 and FLT3 are recurring genetic alternations in AML and related to treatment choice, minimal residual disease monitoring and prognosis prediction. We found that the expression levels of siglec1, CD22, siglec14 were significantly down-regulated whereas the expression levels of CD33 and siglec15 were significantly up-regulated in AML patients with FLT3 mutation. In addition, siglec1, CD22, MAG, siglec6, siglec8 showed reduced expression levels while siglec7, siglec10 and siglec16 exhibited increased expression in AML patients with NPM1 mutation. In AML patients with IDH1 R132 mutation, the expression levels of siglec6 and siglec9 were significantly lower than those without this mutation.

### Immune infiltration in AML patients with different siglec expression

**Fig. 3:**
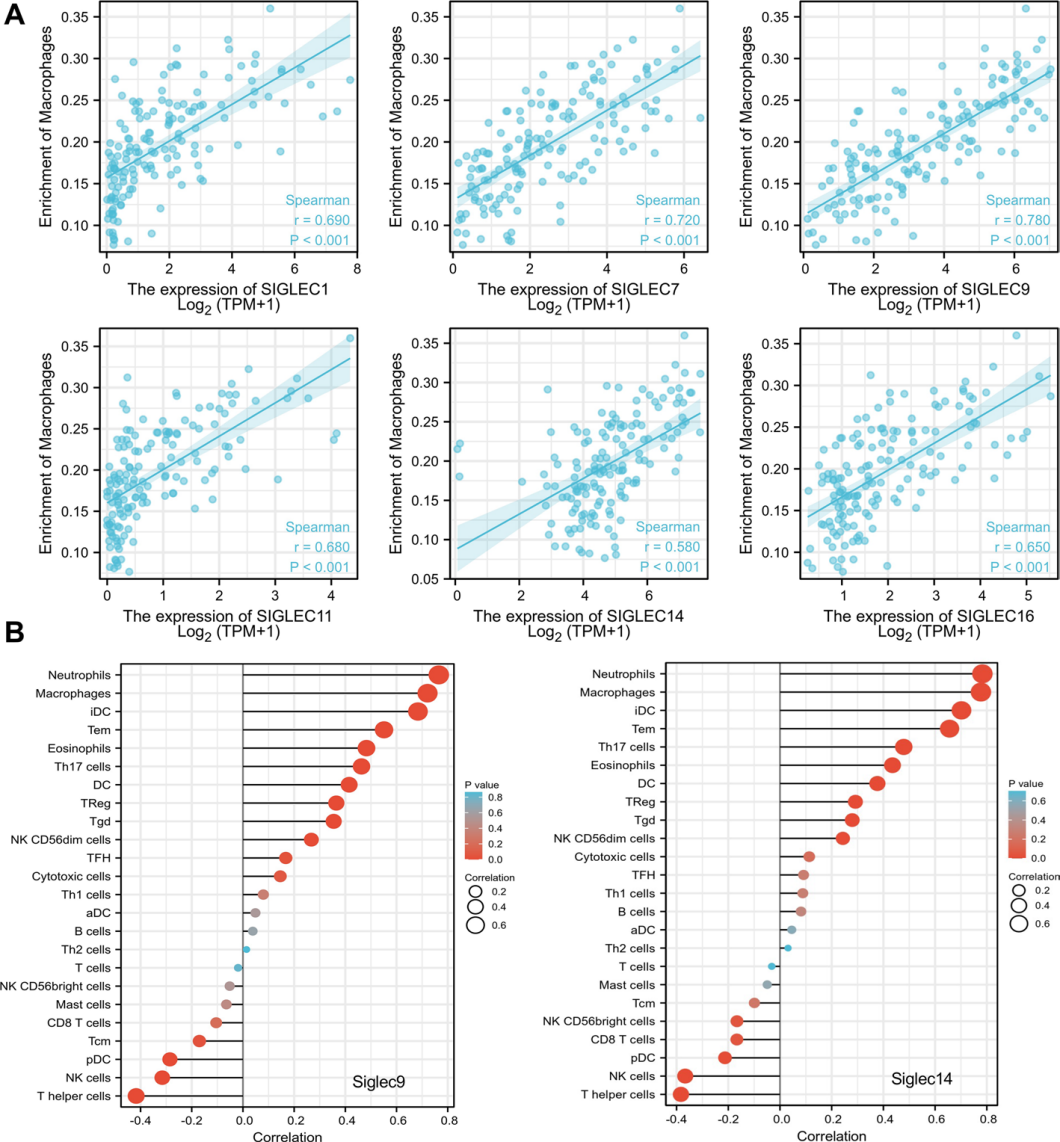
The correlation between immune cell infiltration and siglec expression. **A** The expression levels of siglec1, siglec7, siglec9, siglec11, siglec14 and siglec16 were positively correlated with the infiltration of macrophages. **B** The correlation of siglec9 and siglec14 expression with the infiltration of different immune cells

Dysregulated immune function is a hallmark of carcinogenesis and siglecs are critical regulators of the immune microenvironment, so we analyzed the correlation between immune infiltration and siglec expression in AML patients. Macrophages/monocytes express a variety of siglecs, including siglec1, CD33, siglec5, siglec7, siglec9, siglec10, siglec11, siglec14, siglec15 and siglec16 [2]. Studies have shown that macrophages have both tumor-promoting and inhibiting roles. Interestingly, we found the infiltration of macrophages was positively correlated with the expression levels of siglec1, siglec7, siglec9, siglec11, siglec14, and siglec16 (*p* < 0.001, r > 0.5) (Fig. 3A). The top four positively correlated immune cell types were the same for siglec1, siglec7, siglec9, siglec11, siglec14, and siglec16: neutrophils, macrophages, iDC and Tem, which indicates there might be common immune regulation mechanisms of these siglecs. The infiltration pattern of various immune cells in correlation with siglec9 and siglec14 expression exemplified the potential role of siglecs in regulating the immune landscape of AML (Fig. 3B).

### Correlation between drug resistance and siglec expression

**Fig. 4:**
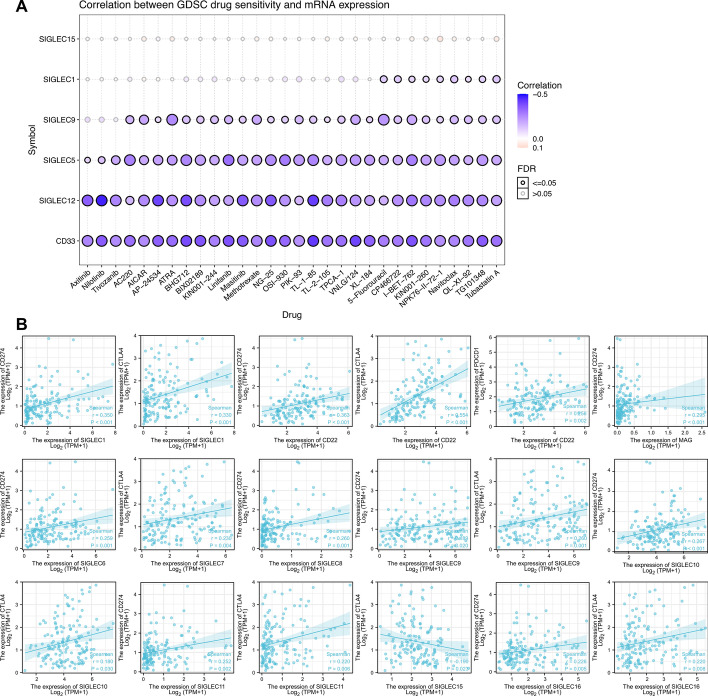
The correlation between treatment resistance and siglec expression. **A**The correlation between drug sensitivity and expression levels of different siglecs based on analysis of the database: Genomics of Drug Sensitivity in Cancer (GDSC). **B** The correlation between the expression levels of siglecs and immune checkpoints, including PDCD1 (PD-1), CD274 (PD-L1) and CTLA4

Evaluation of patient’s drug responsiveness and monitoring drug resistance are of critical importance during AML treatment. We screened the GDSC database and identified the correlations between drug sensitivity and siglec expression (Fig. 4A). Among the screened drugs, all-trans retinoic acid (ATRA) is a well-established agent to induce the differentiation of leukemic promyelocytes in the treatment of acute promyelocytic leukemia [29]. Our results showed that the sensitivity to ATRA was negatively correlated with the expression of CD33, siglec5, siglec9 and siglec12 (FDR ≤ 0.05). AC220 is a selective inhibitor of FLT3 [30] and is shown to be effective in relapsed or refractory AML with FLT3-ITD mutation [31]. We found that the expression levels of CD33, siglec5, siglec9 and siglec12 were negatively correlated with the sensitivity to AC220 (FDR ≤ 0.05). Another inhibitor of FLT3-ITD-driven AML is AP24534 (ponatinib), which inhibits FLT3 activity and induces leukemia cell apoptosis [32]. The sensitivity to AP24534 was negatively correlated with the mRNA expression of CD33, siglec5, and siglec12.

Immune checkpoint inhibitors are emerging as novel and potent cancer therapeutics. However, the efficacy of immune checkpoint therapy is restricted by the expression of immune checkpoints, including PDCD1 (PD-1), CD274 (PD-L1) and CTLA4. Therefore, we explored the relationship between the expression of siglecs and immune checkpoints (Fig. 4B). We found that PDCD1 expression was positively correlated with the expression of CD22. CD274 expression was positively correlated with the expression of siglec1, CD22, MAG, siglec6, siglec8, siglec9, siglec10, siglec11 and siglec16. The expression of CTLA4 was positively correlated with the expression of siglec1, CD22, siglec7, siglec9, siglec10, siglec11, and siglec16. Intriguingly, only the expression of siglec15 was negatively correlated with CTLA4 expression.

### Receiver operating characteristic curve analysis of siglecs

**Fig. 5:**
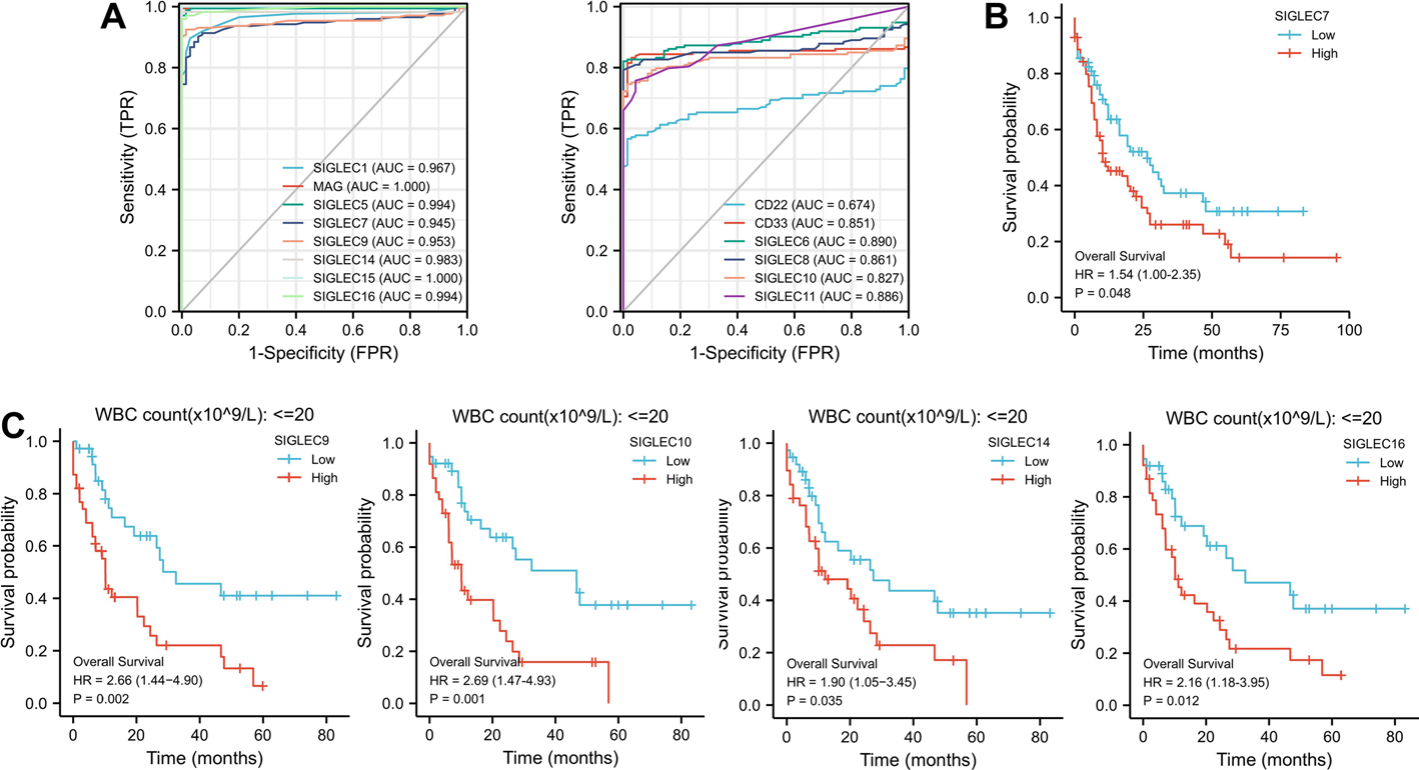
The receiver operating characteristic curve analysis (**A**), Kaplan-Meier survival analysis (**B**), and subgroup Kaplan-Meier curves (**C**) of the siglec family in AML. False positive rate, FPR; true positive rate, TPR

To determine diagnostic value of siglecs in AML, we plotted ROC curve using the AML patient data form TCGA and healthy control data from GTEx database (Fig. 5A). We found that siglec15 (AUC = 1.000, CI 1.000–1.000) and MAG (AUC = 1.000, CI 0.999–1.000) had the highest diagnostic accuracy in AML, followed by siglec16 (AUC = 0.994, CI 0.988–1.000), siglec5 (AUC = 0.994, CI 0.983–1.000), and siglec14 (AUC = 0.983, CI 0.963–1.000), siglec1 (AUC = 0.967, CI 0.946–0.989), siglec9 (AUC = 0.953, CI 0.925–0.981) and siglec7 (AUC = 0.945, CI 0.916–0.975). These results show that the siglec family has high diagnostic accuracy in AML and may serve as novel diagnostic biomarkers.

### Prognosis analysis

Kaplan–Meier survival analysis was performed to evaluate the relationship between siglec expression and AML patient overall survival (Fig. 5B and C). AML patients were classified into two groups with high and low expression of selected siglec according to the median siglec expression level. We found that the overall survival probability of AML patients was higher in those with lower expression of siglec7 (*p* = 0.048, HR 1.54, CI 1.00–2.35) (Fig. 5B). In sub-group analysis, AML patients with WBC count ≤ 20 × 10^9^/L had significantly reduced overall survival probability when they had higher expression of siglec9 (*p* = 0.002, HR 2.66, CI 1.44–4.90), siglec10 (*p* = 0.001, HR 2.69, CI 1.47–4.93), siglec14 (*p* = 0.035, HR 1.90, CI 1.05–3.45) or siglec16 (*p* = 0.012, HR 2.16, CI 1.18–3.95) (Fig. 5C). Univariate and multivariate COX regression analyses were also performed to evaluate the prognostic value of siglec family members in AML (Additional file 3: Table S3). Multivariate regression analysis showed that siglec9 (*p* = 0.018, HR 1.142, CI 1.023–1.275) and siglec15 (*p* = 0.028, HR 0.735, CI 0.559–0.967) are predictive variables for AML outcome.

### Differentially expressed genes in AML patients with low- and high-expressed siglec9/14

**Fig. 6:**
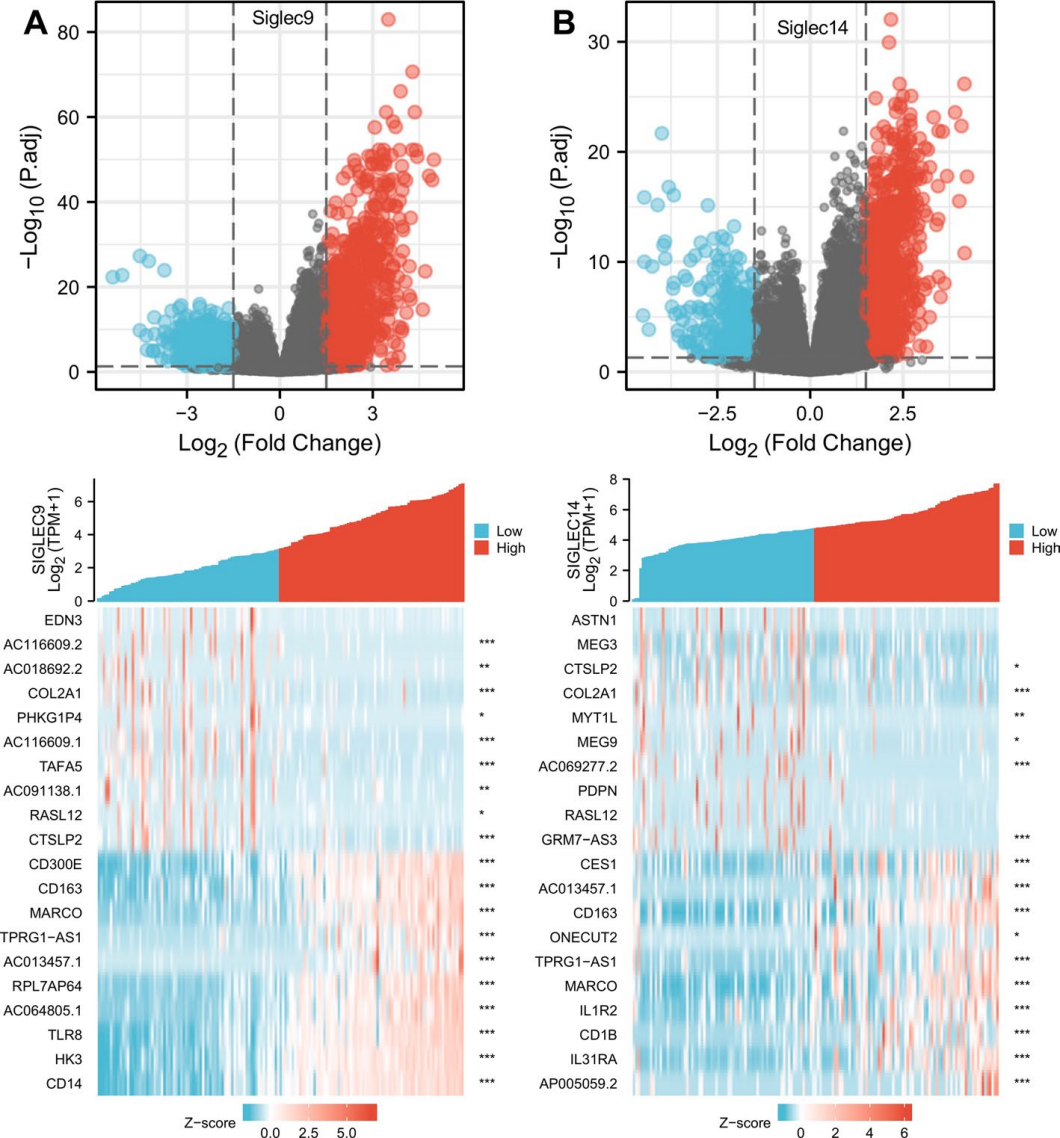
Differential gene expression in AML patients with low- and high- expressed siglec9 and siglec14. The volcano plots showed differentially expressed genes in AML patients with low and high expression of siglec9 (**A**) and siglec14 (**B**). The heatmaps showed the top 10 up-regulated and top 10 down-regulated genes in AML patients with low- and high-expressed siglec9 (**A**) and siglec14 (**B**). *, *p* < 0.05; **, *p* < 0.01; ***, *p* < 0.001

Among the siglec family members, we found that siglec9 and siglec14 were significantly dysregulated when comparing between AML patients and healthy controls, correlated with various clinical features, associated with macrophage infiltration, of high diagnostic value in ROC curve, and of prognostic power in sub-group Kaplan–Meier survival analysis. Therefore, we selected siglec9 and siglec14 for differential gene expression analysis. When comparing AML patients with low- and high-expressed siglec9, there were a total of 1523 genes that were significantly differentially expressed (|log2(FC)|> 1.5 and p.adj < 0.05), with 1021 genes up-regulated and 502 genes down-regulated in the high siglec9 expression group (Fig. 6A). For AML patients with low- and high-expressed siglec14, there were a total of 1086 significantly differentially expressed genes (|log2(FC)|> 1.5 and p.adj < 0.05), including 757 up-regulated genes and 329 down-regulated genes (Fig. 6B). The 10 most up-regulated and down-regulated genes for each comparison were plotted in heatmaps (Fig. 6, bottom panel). Spearman correlation analysis was performed for each of the DEG with the corresponding siglec, and significant pairs were noted on the right side of heatmaps. Intriguingly, we found that podoplanin (PDPN) was significantly up-regulated in AML patients with low expression of siglec14 (FC = − 4.46, p.adj = 9.82 × 10^–11^). Though not shown on the heatmap, podoplanin was similarly significantly up-regulated in AML patients with low expression of siglec9 (FC = − 4.04, p.adj = 1.18 × 10^–9^). Physiologically, podoplanin is a lymphatic endothelial cell marker that is not expressed in blood cells or blood vessels. However, podoplanin is found to be up-regulated in the leukemic promyelocytes of acute promyelocytic leukemia, which causes aberrant platelet binding, activation and aggregation [33]. This supports the great value of the DEG identified in our study, which might be potential biomarkers in AML diagnosis and treatment.

### Functional enrichment analysis

**Fig. 7:**
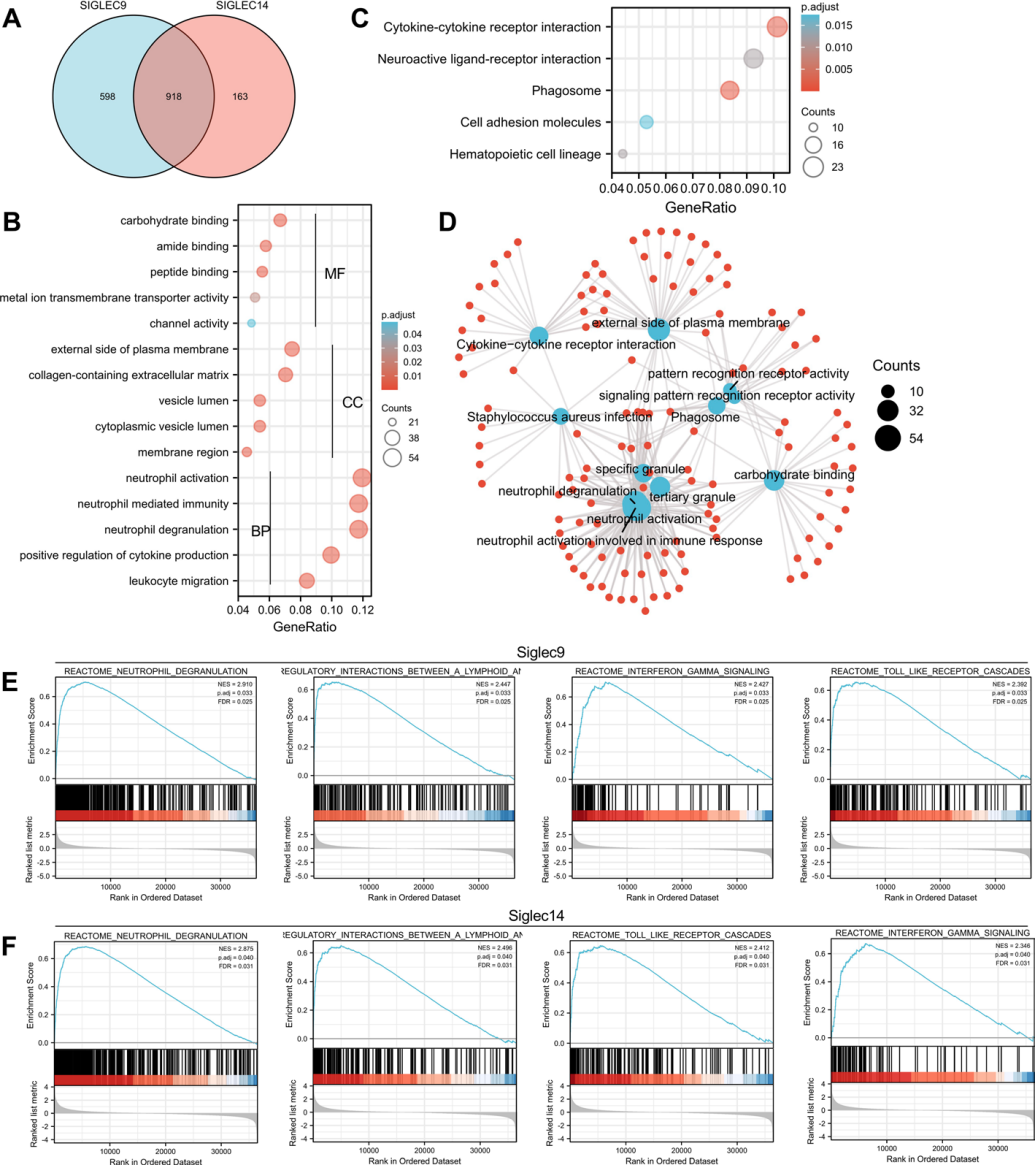
Functional enrichment analysis. **A** Venn plot of differentially expressed genes (DEG) in AML patients with low- and high-expressed siglec9 and siglec14. **B** Bubble plot showing representative enriched GO terms (5 on top: MF; 5 in middle: CC; 5 on bottom: BP). The bubble size represents the number of enriched genes, whereas the bubble color represents p.adj. **C** Bubble plot showing representative enriched KEGG pathways. **D** Visualization of highly enriched GO/KEGG terms and associated genes. The blue node represents GO/KEGG term, whereas the red node represents specific molecule. The node size represents the number of enriched genes. Enriched gene sets revealed by GSEA analysis in AML patients with low- and high-expressed siglec9 (**E**) and siglec14 (**F**) were shown

The DEG in AML patients with low- and high-expressed siglec9 and low- and high-expressed siglec14 were merged and there were 918 shared genes (Fig. 7A). The top 300 most differentially expressed genes among the shared genes were subject to GO and KEGG analyses. For GO analysis, there were 457 BP terms, 46 CC terms and 58 MF terms that were significantly enriched (p.adj < 0.05 and qvalue < 0.2). Meanwhile, there were 17 significantly enriched KEGG pathways (p.adj < 0.05 and qvalue < 0.2). The representative, highly enriched BP, MF and CC terms were visualized in bubble plots (Fig. 7B). Interestingly, these neutrophil-related BP terms were significantly enriched: neutrophil activation, neutrophil degranulation, neutrophil mediated immunity, positive regulation of cytokine production and leukocyte migration. The representative highly enriched KEGG pathways included cytokine-cytokine receptor interaction, neuroactive ligand-receptor interaction, phagosome, cell adhesion molecules, and hematopoietic cell lineage (Fig. 7C). The highly enriched GO/KEGG terms and associated genes were co-visualized (Fig. 7D).

All genes with corresponding log2(FC) values were used as input for GSEA analysis (Fig. 7E and F). Interestingly, siglec9 and siglec14 shared high similarity between the highly enriched gene sets, which included reactome neutrophil degranulation, reactome immunoregulatory interactions between a lymphoid and a non-lymphoid cell, reactome toll like receptor cascades, and reactome interferon gamma signaling.

### Siglec-based and aging-related 9-gene signature for AML outcome prediction

**Fig. 8:**
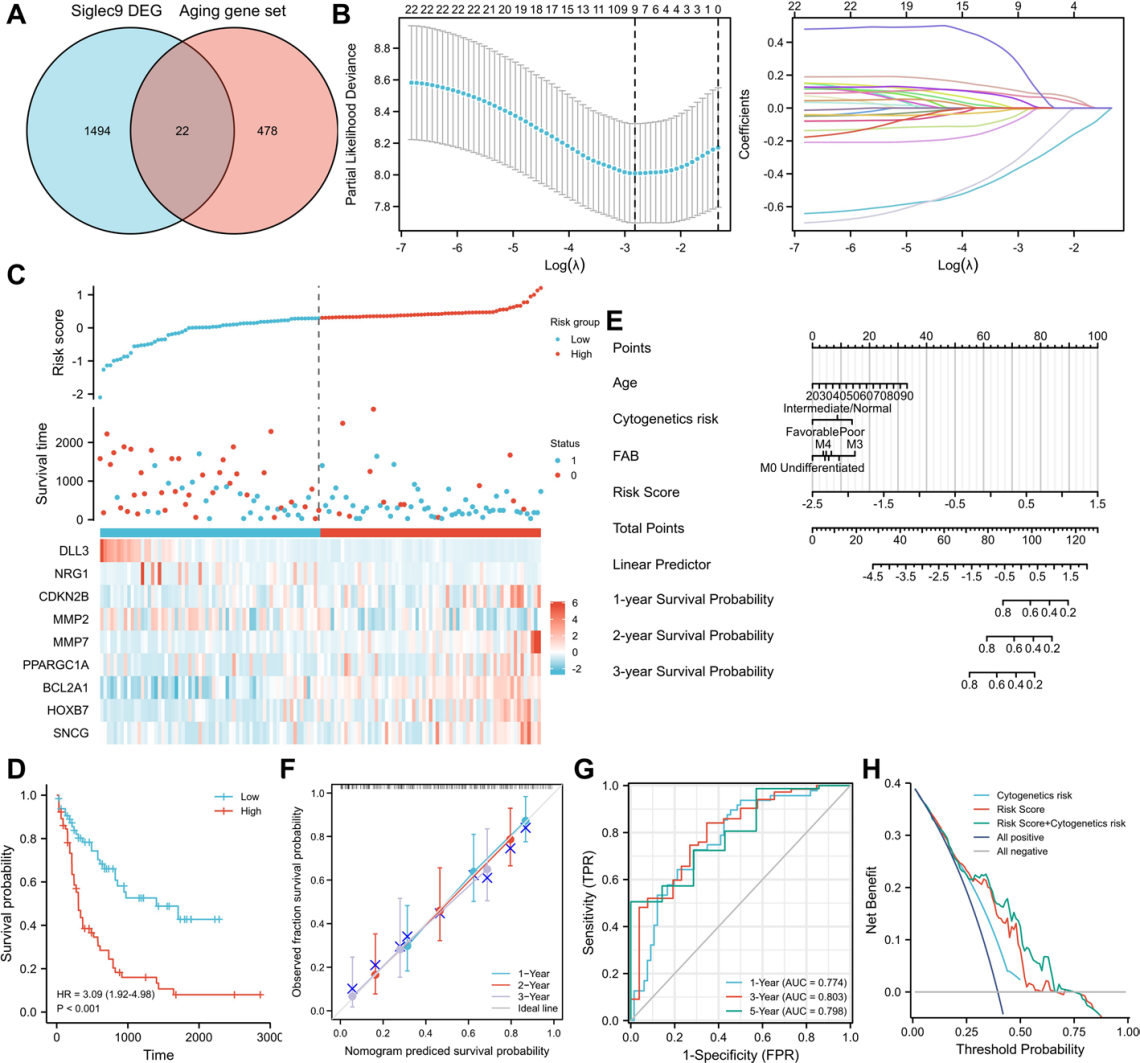
Establishment of the siglec-based and aging-related 9-gene prediction model. **A** Lasso regression analysis of the 22 candidate genes. **B** Lasso variable trace plot. **C** Risk score was calculated and plotted for each AML patient based on the 9-gene prediction model. **D** Survival probability of AML patients with high and low risk scores calculated using the 9-gene prediction model. Nomogram (**E**) and calibration graph (**F**) showing the prediction of AML patient outcome according to age, cytogenetics risk, FAB and risk score. **G** The time-dependent receiver operating characteristic curve of the prediction model. False positive rate, FPR; true positive rate, TPR. **H** DCA plot showing net benefit generated by the 9-gene prediction model

The aging-related gene set was downloaded from the National Genomics Data Center [20]. The DEG identified between AML patients with low- and high-expressed siglec9 were merged with the aging-related gene set (Fig. 8A). A total of 22 merged genes were identified and subjected to lasso regression with tenfold cross validation. Lasso regression analysis revealed 9 genes for building the aging-related prediction model, which were: DLL3, NRG1, CDKN2B, MMP2, PPARGC1A, HOXB7, SNCG, MMP7, and BCL2A1, with their regression coefficients being − 0.333516269, − 0.255912758, − 0.006004825, − 0.006263545, − 0.037486339, 0.026886551, 0.182960345, 0.093034382, and 0.067815218, respectively. Lasso variable trace plot was also plotted to visualize the lasso coefficients (Fig. 8B). The risk score was calculated and plotted for each AML patient using the above prediction model (Fig. 8C).

Next, we categorized the AML patients into two groups according to median risk score. AML patients with higher risk scores had significantly reduced survival probability (*p* < 0.001, HR 3.09, CI 1.92–4.98) (Fig. 8D). Age, cytogenetics risk, FAB and risk score calculated based on the 9-gene model were used to perform nomogram analysis for the prediction of 1-year, 2-year and 3-year survival of AML patients (Fig. 8E). The calibration graph showed that the nomogram predicted survival probability and observed fraction survival probability were highly consistent (Fig. 8F). Time-dependent ROC curve analysis was performed, and the results revealed that the AUC of 1-year, 3-year and 5-year survival were 0.774, 0.803 and 0.798, respectively, indicating the high predictive ability of our novel 9-gene prognostic model (Fig. 8G). Clinically, cytogenetics risk assessment categorizes AML patients into favorable, intermediate/normal, and poor groups for prognosis prediction. We performed decision curve analysis (DCA) and showed that the prediction accuracy of our siglec- and aging-related 9-gene prediction model was higher than cytogenetics risk assessment and the combination of both would generate even more net benefit during AML patient outcome prediction (Fig. 8H).

### Evaluation of the novel 9-gene prognostic model using a validation cohort

**Fig. 9:**
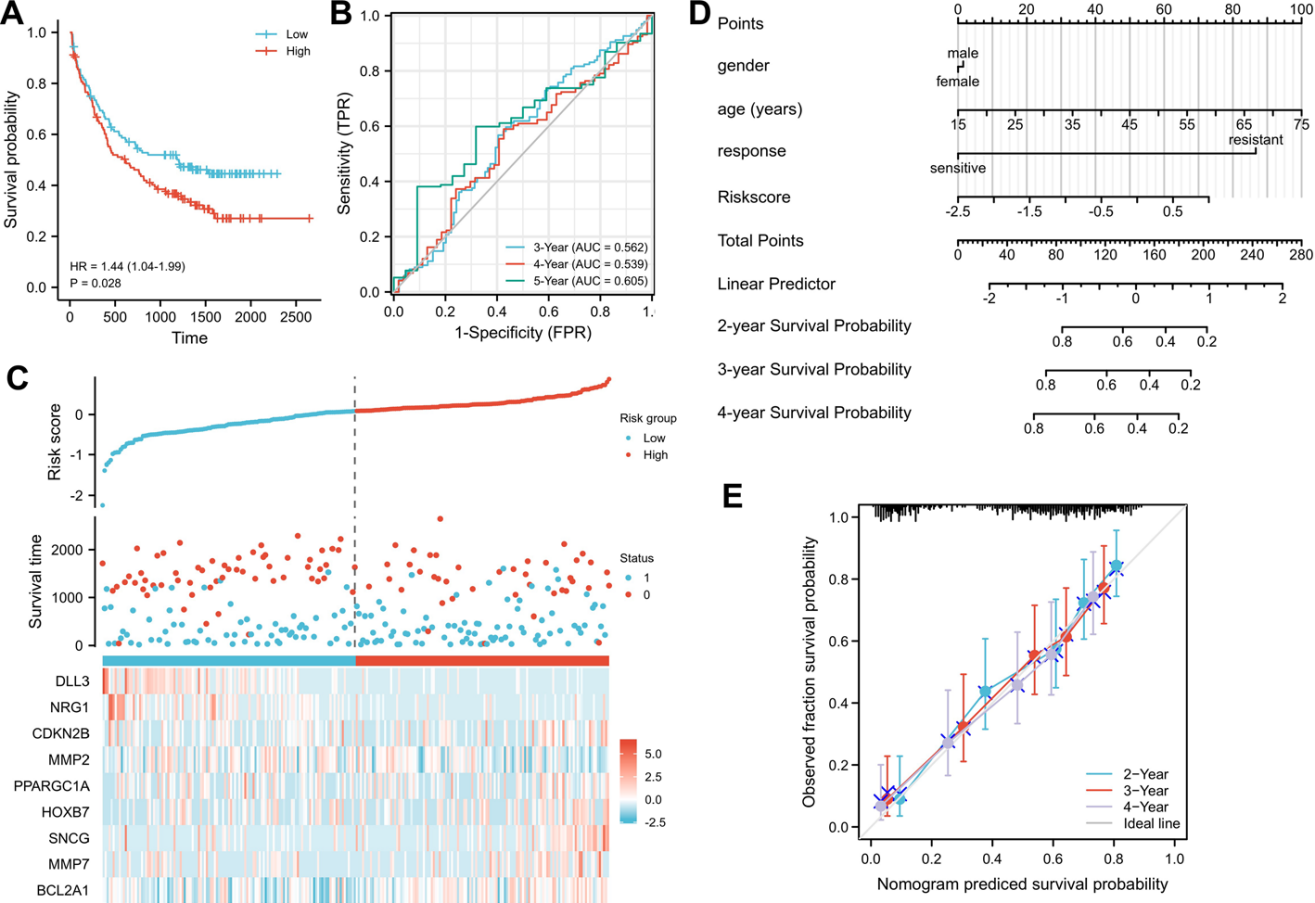
Validation of the novel 9-gene prediction model. **A** Survival probability of AML patients from the validation cohort with high and low risk scores calculated using the 9-gene prediction model. **B** The time-dependent receiver operating characteristic curve of the prediction model in the validation cohort. False positive rate, FPR; true positive rate, TPR. **C** Risk score was calculated using the 9-gene prediction model and plotted for each AML patient from the validation cohort. **D** Nomogram showing the prediction of AML patient outcome according to gender, age, treatment resistance and risk score. **E** Calibration graph showing the consistency between nomogram predicted survival probability and observed fraction survival probability

To test the ability of the siglec-based and aging-related 9-gene prognostic model in predicting AML patient outcome in a validation cohort, we download the GSE106291 dataset from GEO database, which consists of 210 patients from the AMLCG-2008 study (NCT01382147) and 40 patients from the AMLG-1999 trial (NCT00266136) [17]. The risk score of each AML patient in this validation cohort was calculated using the expression level of 9 genes and their corresponding regression coefficients. Median risk score categorized the AML patients into two groups with low and high risk scores. Interestingly, AML patients with lower risk scores had significantly higher survival probability whereas those with higher risk scores had significantly reduced survival probability (*p* = 0.028, HR 1.44, CI 1.04–1.99) (Fig. 9A). This showed that our novel 9-gene model could effectively predict patient survival probability in the validation cohort. Time-dependent ROC curve analysis showed that the AUC for 3-year, 4-year and 5-year survival were 0.562, 0.539 and 0.605, respectively (Fig. 9B). The risk score was calculated for each AML patient in the validation cohort using our 9-gene prediction model and the values were plotted (Fig. 9C). Gender, age, treatment response, and risk score were used in nomogram analysis for predicting 2-year, 3-year and 4-year survival probability of AML patients (Fig. 9D). The calibration graph showed that in this validation cohort, there was good consistency between the nomogram predicted survival probability and observed fraction survival probability (**Fig. ****9****E**). These results showed that novel siglec-based and aging-related 9-gene signature exhibited good predicting performance in the validation cohort.

## Discussion

Due to the regulatory function of siglecs in cancer immunity, there has been growing interest in studying the role of siglecs in AML and great progress has been made recently. In this study, we showed that the expression pattern of the siglec family was significantly altered in AML, and it was correlated to AML patient clinical characteristics, immune cell infiltration, drug resistance and survival outcome. CAR-T immunotherapies directed against CD33 and siglec6 have been shown to exhibit anti-leukemia activity in xenograft mouse AML models [4, 6, 34]. The immunoconjugate gemtuzumab is made of gemtuzumab, a monoclonal antibody against CD33, and the coupled ozogamicin for the treatment of AML in human [3]. Recently, bispecific antibodies targeting CD33 IgV and IgC domains have been developed and found to inhibit AML [5]. Siglec-7 mediates immune inhibition by interacting with the mucin-type glycoprotein CD43 on leukemia cells, and CD43 knockout or blockade in leukemia cells disrupted this interaction to enhance anti-tumor immune reaction [9]. Previous studies also indicate that siglec9 is an inhibitory immune checkpoint in tumor [7, 8]. Endogenous immune response and therapeutic efficacy of tumor-targeting antibodies were inhibited in a humanized mouse model for siglec7/9, whereas blocking siglec7/9 using antibodies could enhance antitumor immunity in mice [7]. Siglec-E, the mouse orthologue of human siglec9, regulates ROS metabolism and its deficiency led to accelerated aging and reduced lifespan in mice [15]. As AML incidence increases in people of older age [10], and cellular aging is one of the major risk factors for leukemogenesis [12], it is interesting to study whether siglec9, the human orthologue of mouse siglec-E, is involved in the human aging process and contributes to AML pathogenesis. Therefore, we analyzed the differentially expressed genes in AML patients with low and high expression of siglec9 and performed functional enrichment analysis. Moreover, a siglec-based and aging-related 9-gene prognostic model was built and validated to have good prediction ability in AML.

We found that siglec expression was correlated with immune cell infiltration in AML. Macrophages are an important type of innate immune cells against self and foreign insults, in which a variety of siglecs are expressed. Tumor-associated macrophages (TAM) represent a pivotal regulator of tumor microenvironment and they can be tumor-inhibitory or tumor-promoting due to their great heterogenicity [35]. Here, we found the infiltration of macrophages was positively correlated with the expression levels of siglec1, siglec7, siglec9, siglec11, siglec14, and siglec16 (*p* < 0.001, r > 0.5). The role of macrophages in cancer is being intensively studied and some TAM overexpressing siglecs, such as siglec10, are identified as immune checkpoints [36]. However, the role of macrophages in AML remains inclusive and our study indicates that siglecs may regulate macrophage biology to affect AML outcome.

Due to the frequent mutation and rapid clonal expansion of AML, drug resistance is a critical issue that could lead to relapse and poor prognosis [37]. We analyzed the correlation between drug sensitivity and mRNA expression of siglecs. Among the screened drugs whose efficacy was correlated with siglec expression, some were well-established therapeutics, such as ATRA, while some were under investigation for use in AML patients, such as AP24534 (ponatinib). We found that the expression of CD33, siglec5, and siglec12 was negatively correlated with the sensitivity of all the displayed drugs (FDR ≤ 0.05).

Siglec9 and siglec14 were significantly upregulated in AML patients, correlated with various clinical features, and associated with macrophage infiltration. They showed high diagnostic value in ROC curve analysis, and prognostic capability in sub-group Kaplan–Meier survival analysis. Differential gene expression analysis was performed for AML patients with low- and high-expressed siglec9 and siglec14, and 918 shared genes were identified. Functional enrichment analysis was performed, and we found that neutrophil degranulation is an interesting process that was an enriched BP term in GO analysis and an enriched gene set in GSEA analysis. Neutrophil degranulation affects tumor microenvironment and promotes the growth and progression of solid tumors [38]. Furthermore, neutrophils are emerging as novel therapeutic targets in cancer and some neutrophil-targeting agents are under clinical trial investigation [39]. Our analysis indicates the importance of neutrophil degranulation in AML pathogenesis and further study is needed to dissect the effects of neutrophil degranulation on AML patient outcome.

Hematopoietic cell aging is one of the major risk factors for leukemogenesis [12]. Multiple genomic, epigenomic and transcriptomic alternations are related to cellular aging and could affect AML patient outcome. We merged the differentially expressed genes in AML patients with low and high-expressed siglec9 with the aging-related gene set. The merged genes were analyzed by lasso regression, based on which we built a siglec-based and aging-related 9-gene prognostic model. Interestingly, among the 9 genes included in the model, some have been found to be associated with AML outcome. For example, CDKN2B methylation has negative prognostic impact on newly diagnosed acute promyelocytic leukemia patients [40]. PPARGC1A is related to enhanced mitochondrial DNA copy number and predicts poor prognosis in pediatric AML [41]. This supports the validity of our analysis and model construction. Consistently, in the validation cohort, survival probability analysis showed significantly improved survival outcome in AML patients with lower risk scores calculated using our new prediction model. Time-dependent ROC curve and nomogram analysis showed good predictive performance of our novel model.

## Conclusions

Overall, our study reveals the dysregulated expression of siglec family in AML and its correlation with AML clinical features, immune cell infiltration, drug resistance and survival outcome. Based on the differentially expressed genes in AML patients and the aging-related gene set, we built a siglec-based and aging-related 9-gene prognostic model, which shows good performance in predicting AML patient outcome.

## Supplementary Information

**Additional file 1: Supplementary Table 1.** The aging-related gene set from the National Genomics Data Center.

**Additional file 2: Supplementary Table 2.** Summary of the general information from 151 acute myeloid leukemia patients at the cancer genome atlas database.

**Additional file 3: Supplementary Table 3.** Univariate and multivariate analyses to reveal the prognostic value of the siglec family in acute myeloid leukemia using information from 151 acute myeloid leukemia patients at the cancer genome atlas database.

## Data Availability

The datasets generated and/or analyzed during this study are publicly available in the TCGA database (https://portal.gdc.cancer.gov/), GTEx database (https://gtexportal.org/home/), GEO database (https://www.ncbi.nlm.nih.gov/geo/), and National Genomics Data Center (https://ngdc.cncb.ac.cn/).

## Abbreviations

AML: Acute myeloid leukemia
CAR-T cell: Chimeric antigen receptor T cell
aDC: Activated dendritic cell
iDC: Immature dendritic cell
pDC: Plasmacytoid dendritic cell
Tcm: T central memory
Tem: T effector memory
Tfh: T follicular helper
Tgd: T gamma delta
GSCA: Gene set cancer analysis
GDSC: Genomics of drug sensitivity in cancer
ROC curve: Receiver operating characteristic curve
KM analysis: Kaplan–Meier analysis
DEG: Differentially expressed gene
GO: Gene ontology
KEGG: Kyoto encyclopedia of genes and genomes
BP: Biological process
CC: Cellular component
MF: Molecular function
GSEA: Gene Set Enrichment Analysis
DCA: Decision curve analysis
ATRA: All-trans retinoic acid
TAM: Tumor-associated macrophages
