# Supplementary material for: Identification and validation of a siglec-based and aging-related 9-gene signature for predicting prognosis in acute myeloid leukemia patients
Source: BMC Bioinformatics. 2022 Jul 19;23:284. doi: 10.1186/s12859-022-04841-5 (PMC9295398; doi:10.1186/s12859-022-04841-5)
Supplement: Supplementary file 2 — Additional file 2: Supplementary Table 2. Summary of the general information from 151 acute myeloid leukemia patients at the cancer genome atlas database. [file 12859_2022_4841_MOESM2_ESM.docx]

**Supplementary Table 2.** Summary of acute myeloid leukemia patient information

| Characteristic | levels | Overall |
| --- | --- | --- |
| n |  | 151 |
| Gender, n (%) | Female | 68 (45%) |
|  | Male | 83 (55%) |
| Race, n (%) | Asian | 1 (0.7%) |
|  | Black or African American | 13 (8.7%) |
|  | White | 135 (90.6%) |
| Age, n (%) | <=60 | 88 (58.3%) |
|  | >60 | 63 (41.7%) |
| WBC count(x10^9/L), n (%) | <=20 | 77 (51.3%) |
|  | >20 | 73 (48.7%) |
| BM blasts(%), n (%) | <=20 | 60 (39.7%) |
|  | >20 | 91 (60.3%) |
| PB blasts(%), n (%) | <=70 | 72 (47.7%) |
|  | >70 | 79 (52.3%) |
| Cytogenetic risk, n (%) | Favorable | 31 (20.8%) |
|  | Intermediate | 82 (55%) |
|  | Poor | 36 (24.2%) |
| FAB classifications, n (%) | M0 | 15 (10%) |
|  | M1 | 35 (23.3%) |
|  | M2 | 38 (25.3%) |
|  | M3 | 15 (10%) |
|  | M4 | 29 (19.3%) |
|  | M5 | 15 (10%) |
|  | M6 | 2 (1.3%) |
|  | M7 | 1 (0.7%) |
| Cytogenetics, n (%) | Normal | 69 (51.1%) |
|  | +8 | 8 (5.9%) |
|  | del(5) | 1 (0.7%) |
|  | del(7) | 6 (4.4%) |
|  | inv(16) | 8 (5.9%) |
|  | t(15;17) | 11 (8.1%) |
|  | t(8;21) | 7 (5.2%) |
|  | t(9;11) | 1 (0.7%) |
|  | Complex | 24 (17.8%) |
| FLT3 mutation, n (%) | Negative | 102 (69.4%) |
|  | Positive | 45 (30.6%) |
| IDH1 R132 mutation, n (%) | Negative | 136 (91.3%) |
|  | Positive | 13 (8.7%) |
| IDH1 R140 mutation, n (%) | Negative | 137 (91.9%) |
|  | Positive | 12 (8.1%) |
| IDH1 R172 mutation, n (%) | Negative | 147 (98.7%) |
|  | Positive | 2 (1.3%) |
| RAS mutation, n (%) | Negative | 142 (94.7%) |
|  | Positive | 8 (5.3%) |
| NPM1 mutation, n (%) | Negative | 117 (78%) |
|  | Positive | 33 (22%) |
| OS event, n (%) | Alive | 54 (35.8%) |
|  | Dead | 97 (64.2%) |
