# Supplementary material for: Identification and validation of a siglec-based and aging-related 9-gene signature for predicting prognosis in acute myeloid leukemia patients
Source: BMC Bioinformatics. 2022 Jul 19;23:284. doi: 10.1186/s12859-022-04841-5 (PMC9295398; doi:10.1186/s12859-022-04841-5)
Supplement: Supplementary file 3 — Additional file 3: Supplementary Table 3. Univariate and multivariate analyses to reveal the prognostic value of the siglec family in acute myeloid leukemia using information from 151 acute myeloid leukemia patients at the cancer genome atlas database. [file 12859_2022_4841_MOESM3_ESM.docx]

**Supplementary Table 3.** Univariate and multivariate analyses of the siglec family in acute myeloid leukemia

| Characteristics | Total(N) | Univariate analysis | |  | Multivariate analysis | |
| --- | --- | --- | --- | --- | --- | --- |
|  |  | Hazard ratio (95% CI) | P value |  | Hazard ratio (95% CI) | P value |
| SIGLEC1 | 140 | 1.105 (0.979-1.247) | 0.105 |  |  |  |
| CD22 | 140 | 1.120 (0.928-1.352) | 0.238 |  |  |  |
| CD33 | 140 | 1.008 (0.835-1.217) | 0.931 |  |  |  |
| MAG | 140 | 1.354 (0.868-2.110) | 0.181 |  |  |  |
| SIGLEC5 | 140 | 1.028 (0.844-1.251) | 0.786 |  |  |  |
| SIGLEC6 | 140 | 1.046 (0.902-1.214) | 0.550 |  |  |  |
| SIGLEC7 | 140 | 1.111 (0.970-1.272) | 0.128 |  |  |  |
| SIGLEC8 | 140 | 0.868 (0.623-1.211) | 0.405 |  |  |  |
| SIGLEC9 | 140 | 1.105 (0.994-1.228) | 0.065 |  | 1.142 (1.023-1.275) | **0.018** |
| SIGLEC10 | 140 | 1.141 (0.970-1.342) | 0.112 |  |  |  |
| SIGLEC11 | 140 | 1.142 (0.922-1.416) | 0.223 |  |  |  |
| SIGLEC14 | 140 | 1.086 (0.940-1.255) | 0.265 |  |  |  |
| SIGLEC15 | 140 | 0.809 (0.631-1.037) | 0.094 |  | 0.735 (0.559-0.967) | **0.028** |
| SIGLEC16 | 140 | 1.134 (0.958-1.343) | 0.143 |  |  |  |
